# Supplementary material for: Meta-Analysis and Gene Set Enrichment Relative to ER Status Reveal Elevated Activity of MYC and E2F in the “Basal” Breast Cancer Subgroup
Source: PLoS One. 2009 Mar 9;4(3):e4710. doi: 10.1371/journal.pone.0004710 (PMC2650420; doi:10.1371/journal.pone.0004710)
Supplement: Dataset S1 — GSEA results from first screen. The file contains a zipped archive of the HTML results of the second GSEA screen. Reports for enriched or depleted datasets may be accessed from the “index.html” link within the my_analysis.GseaPreranked.1219975950625.rpt.zip ZIP archive. Enrichment plots are available for each of the 50 most enriched and depleted gene sets. (13.32 MB ZIP) [file pone.0004710.s001.zip › my_analysis.GseaPreranked.1219975950625.rpt.zip!//SGCGSSAAA_V$E2F1DP2_01.html]

Details for gene set SGCGSSAAA\_V$E2F1DP2\_01[GSEA]

|  || Dataset | zSco.ERneg.to.ERpos\_collapsed |
| Phenotype | NoPhenotypeAvailable |
| Upregulated in class | na\_pos |
| GeneSet | SGCGSSAAA\_V$E2F1DP2\_01 |
| Enrichment Score (ES) | 0.5390253 |
| Normalized Enrichment Score (NES) | 2.271137 |
| Nominal p-value | 0.0 |
| FDR q-value | 0.0 |
| FWER p-Value | 0.0 |
Table: GSEA Results Summary

  

Fig 1: Enrichment plot: SGCGSSAAA\_V$E2F1DP2\_01      
 Profile of the Running ES Score & Positions of GeneSet Members on the Rank Ordered List

  

| PROBE | GENE SYMBOL | GENE\_TITLE | RANK IN GENE LIST | RANK METRIC SCORE | RUNNING ES | CORE ENRICHMENT || 1 | E2F3 |  |  | 6 | 7.369 | 0.0278 | Yes |
| 2 | NASP |  |  | 59 | 6.201 | 0.0476 | Yes |
| 3 | PIM1 |  |  | 78 | 6.025 | 0.0693 | Yes |
| 4 | AK2 |  |  | 85 | 5.971 | 0.0918 | Yes |
| 5 | GPRC5B |  |  | 103 | 5.845 | 0.1129 | Yes |
| 6 | MCM4 |  |  | 180 | 5.436 | 0.1279 | Yes |
| 7 | MCM6 |  |  | 198 | 5.317 | 0.1470 | Yes |
| 8 | GMNN |  |  | 206 | 5.302 | 0.1668 | Yes |
| 9 | HMGA1 |  |  | 244 | 5.125 | 0.1837 | Yes |
| 10 | CDC25A |  |  | 281 | 4.993 | 0.2001 | Yes |
| 11 | MCM7 |  |  | 289 | 4.970 | 0.2186 | Yes |
| 12 | RANBP1 |  |  | 380 | 4.733 | 0.2299 | Yes |
| 13 | SYNCRIP |  |  | 402 | 4.679 | 0.2462 | Yes |
| 14 | SNRPD1 |  |  | 440 | 4.552 | 0.2609 | Yes |
| 15 | SUV39H1 |  |  | 495 | 4.361 | 0.2735 | Yes |
| 16 | TYRO3 |  |  | 516 | 4.316 | 0.2885 | Yes |
| 17 | RPS20 |  |  | 615 | 4.101 | 0.2968 | Yes |
| 18 | MCM2 |  |  | 630 | 4.080 | 0.3114 | Yes |
| 19 | MCM3 |  |  | 656 | 4.019 | 0.3249 | Yes |
| 20 | NCL |  |  | 678 | 3.977 | 0.3386 | Yes |
| 21 | ILF3 |  |  | 801 | 3.774 | 0.3438 | Yes |
| 22 | MSH2 |  |  | 819 | 3.748 | 0.3569 | Yes |
| 23 | MYC |  |  | 1025 | 3.447 | 0.3545 | Yes |
| 24 | UNG |  |  | 1070 | 3.390 | 0.3642 | Yes |
| 25 | FANCC |  |  | 1175 | 3.264 | 0.3688 | Yes |
| 26 | NOLC1 |  |  | 1222 | 3.217 | 0.3777 | Yes |
| 27 | PRKDC |  |  | 1234 | 3.204 | 0.3891 | Yes |
| 28 | DNAJC9 |  |  | 1243 | 3.198 | 0.4008 | Yes |
| 29 | POLD1 |  |  | 1260 | 3.182 | 0.4118 | Yes |
| 30 | FBXO5 |  |  | 1304 | 3.137 | 0.4205 | Yes |
| 31 | SFRS10 |  |  | 1320 | 3.118 | 0.4313 | Yes |
| 32 | PCNA |  |  | 1346 | 3.087 | 0.4413 | Yes |
| 33 | ING3 |  |  | 1398 | 3.042 | 0.4491 | Yes |
| 34 | EFNA5 |  |  | 1488 | 2.953 | 0.4536 | Yes |
| 35 | ATAD2 |  |  | 1532 | 2.917 | 0.4616 | Yes |
| 36 | NUP62 |  |  | 1684 | 2.770 | 0.4607 | Yes |
| 37 | E2F1 |  |  | 1695 | 2.764 | 0.4706 | Yes |
| 38 | HIRA |  |  | 1696 | 2.763 | 0.4812 | Yes |
| 39 | PKMYT1 |  |  | 1715 | 2.753 | 0.4904 | Yes |
| 40 | FKBP5 |  |  | 1729 | 2.743 | 0.4999 | Yes |
| 41 | PLAGL1 |  |  | 1762 | 2.718 | 0.5079 | Yes |
| 42 | UGCGL1 |  |  | 1779 | 2.709 | 0.5170 | Yes |
| 43 | CASP8AP2 |  |  | 1930 | 2.587 | 0.5156 | Yes |
| 44 | RRM2 |  |  | 2047 | 2.485 | 0.5163 | Yes |
| 45 | H2AFZ |  |  | 2105 | 2.447 | 0.5214 | Yes |
| 46 | SFRS7 |  |  | 2226 | 2.370 | 0.5214 | Yes |
| 47 | POLR2A |  |  | 2274 | 2.334 | 0.5268 | Yes |
| 48 | FANCG |  |  | 2282 | 2.332 | 0.5352 | Yes |
| 49 | GLRA3 |  |  | 2348 | 2.287 | 0.5390 | Yes |
| 50 | HNRPD |  |  | 2585 | 2.156 | 0.5294 | No |
| 51 | PTMA |  |  | 2647 | 2.119 | 0.5329 | No |
| 52 | DNMT1 |  |  | 2844 | 2.017 | 0.5258 | No |
| 53 | ATF5 |  |  | 2941 | 1.962 | 0.5261 | No |
| 54 | NR6A1 |  |  | 3000 | 1.931 | 0.5291 | No |
| 55 | CDC6 |  |  | 3185 | 1.847 | 0.5222 | No |
| 56 | C10ORF119 |  |  | 3202 | 1.838 | 0.5280 | No |
| 57 | PAQR4 |  |  | 3434 | 1.736 | 0.5172 | No |
| 58 | PRPF4B |  |  | 3435 | 1.736 | 0.5239 | No |
| 59 | SMAD6 |  |  | 3436 | 1.736 | 0.5305 | No |
| 60 | TOPBP1 |  |  | 3475 | 1.722 | 0.5342 | No |
| 61 | MSH5 |  |  | 3656 | 1.639 | 0.5269 | No |
| 62 | PRPS1 |  |  | 3889 | 1.556 | 0.5153 | No |
| 63 | POLE2 |  |  | 3975 | 1.520 | 0.5147 | No |
| 64 | STAG2 |  |  | 4224 | 1.421 | 0.5013 | No |
| 65 | RQCD1 |  |  | 4253 | 1.412 | 0.5046 | No |
| 66 | HNRPUL1 |  |  | 4310 | 1.387 | 0.5057 | No |
| 67 | CLSPN |  |  | 4483 | 1.324 | 0.4977 | No |
| 68 | GABRB3 |  |  | 4549 | 1.303 | 0.4978 | No |
| 69 | KIAA0101 |  |  | 4770 | 1.230 | 0.4859 | No |
| 70 | HNRPR |  |  | 4913 | 1.185 | 0.4797 | No |
| 71 | HTF9C |  |  | 4986 | 1.161 | 0.4787 | No |
| 72 | ARHGAP6 |  |  | 4991 | 1.159 | 0.4828 | No |
| 73 | MYH10 |  |  | 5076 | 1.124 | 0.4807 | No |
| 74 | HIST1H4A |  |  | 5241 | 1.067 | 0.4724 | No |
| 75 | EED |  |  | 5267 | 1.060 | 0.4746 | No |
| 76 | ACO2 |  |  | 5713 | 0.907 | 0.4444 | No |
| 77 | ADAMTS2 |  |  | 5988 | 0.817 | 0.4268 | No |
| 78 | YBX2 |  |  | 6299 | 0.716 | 0.4060 | No |
| 79 | ALDH6A1 |  |  | 6317 | 0.711 | 0.4075 | No |
| 80 | C20ORF67 |  |  | 6482 | 0.657 | 0.3976 | No |
| 81 | H2AFV |  |  | 6569 | 0.627 | 0.3935 | No |
| 82 | POLD3 |  |  | 6667 | 0.601 | 0.3884 | No |
| 83 | TFAP4 |  |  | 6777 | 0.561 | 0.3823 | No |
| 84 | AP1S1 |  |  | 7316 | 0.377 | 0.3430 | No |
| 85 | GATA1 |  |  | 7431 | 0.334 | 0.3357 | No |
| 86 | MGC13053 |  |  | 7620 | 0.261 | 0.3224 | No |
| 87 | SLC9A5 |  |  | 8303 | 0.000 | 0.2708 | No |
| 88 | MXD3 |  |  | 8312 | -0.002 | 0.2702 | No |
| 89 | HIST1H2BK |  |  | 8356 | -0.020 | 0.2670 | No |
| 90 | HMG2L1 |  |  | 8412 | -0.042 | 0.2630 | No |
| 91 | SYNGR4 |  |  | 8638 | -0.147 | 0.2465 | No |
| 92 | FMO4 |  |  | 8775 | -0.199 | 0.2370 | No |
| 93 | SFRS1 |  |  | 8891 | -0.255 | 0.2293 | No |
| 94 | DCK |  |  | 8935 | -0.280 | 0.2271 | No |
| 95 | ID3 |  |  | 8993 | -0.308 | 0.2240 | No |
| 96 | PPM1D |  |  | 9078 | -0.350 | 0.2189 | No |
| 97 | PCSK1 |  |  | 9351 | -0.483 | 0.2002 | No |
| 98 | MAZ |  |  | 9415 | -0.521 | 0.1974 | No |
| 99 | HIST1H3A |  |  | 9503 | -0.563 | 0.1930 | No |
| 100 | ZCCHC8 |  |  | 9511 | -0.566 | 0.1946 | No |
| 101 | ASXL2 |  |  | 9736 | -0.689 | 0.1803 | No |
| 102 | ZCWPW1 |  |  | 9773 | -0.709 | 0.1803 | No |
| 103 | MRPL40 |  |  | 10273 | -1.004 | 0.1464 | No |
| 104 | XTP3TPA |  |  | 10592 | -1.236 | 0.1270 | No |
| 105 | C11ORF30 |  |  | 10656 | -1.284 | 0.1272 | No |
| 106 | C14ORF130 |  |  | 10945 | -1.523 | 0.1112 | No |
| 107 | AP4M1 |  |  | 11123 | -1.683 | 0.1043 | No |
| 108 | CSPG2 |  |  | 11207 | -1.748 | 0.1047 | No |
| 109 | PHF15 |  |  | 11215 | -1.755 | 0.1109 | No |
| 110 | HOXC10 |  |  | 11345 | -1.878 | 0.1083 | No |
| 111 | FHOD1 |  |  | 11408 | -1.941 | 0.1110 | No |
| 112 | USP52 |  |  | 11931 | -2.478 | 0.0810 | No |
| 113 | CXORF12 |  |  | 12144 | -2.762 | 0.0755 | No |
| 114 | RET |  |  | 12638 | -3.506 | 0.0516 | No |
Table: GSEA details [plain text format]

  

Fig 2: SGCGSSAAA\_V$E2F1DP2\_01: Random ES distribution      
 Gene set null distribution of ES for **SGCGSSAAA\_V$E2F1DP2\_01**
